# An Able-Bodied Study for Potential Usage of a Knee Scooter as a Constraint-Induced Movement Therapy (CIMT) Gait Training Device

**DOI:** 10.3390/jfmk9010045

**Published:** 2024-03-04

**Authors:** Jaewook Kim, Yekwang Kim, Juhui Moon, Seung-Jong Kim

**Affiliations:** Department of Biomedical Engineering, Korea University College of Medicine, Seoul 02841, Republic of Korea; jaewook_kim@korea.ac.kr (J.K.); rladprhkd12@korea.ac.kr (Y.K.); answn520@korea.ac.kr (J.M.)

**Keywords:** biomechanics, gait, knee scooter, kinematics, electromyography, CIMT

## Abstract

Post-stroke gait is characterized by slow and asymmetrical hemiparetic gait. This is attributed to the paretic lower limb which has limited plantar propulsion. The most effective method to restore paretic limb function is constraint-induced movement therapy (CIMT), which promotes the usage of the paretic limb by restricting the movement of the unafflicted limb. However, due to the necessity of both lower limbs to perform gait, CIMT methods could not be directly applied for gait rehabilitation. In this study, we explore the feasibility of utilizing a knee scooter as a means to facilitate CIMT gait training. We hypothesize that if lower limb kinematics and muscle activation patterns during gait with a knee scooter match that of natural gait, the knee scooter could be utilized for CIMT gait training. We measured the lower-limb joint angles, plantar force, *EMG* patterns, stride length, and step times of 13 healthy subjects during gait with a knee scooter and natural gait. The results suggest that the gait patterns while using the knee scooter closely resemble those of natural gait.

## 1. Introduction

Bipedal gait, which is directly related with quality of life, is a key factor in achieving independence [1,2,3]. It not only serves as a highly energy-efficient mode of ambulation but also as a basis to carry out a diverse array of tasks in everyday activities [4,5,6,7,8]. However, individuals who suffer from post-stroke hemiparesis are characterized by slow and asymmetrical gait [9,10,11,12,13,14]. This is due to the impaired function of the paretic limb which limits the propulsive force necessary for forward propulsion [15].

On a positive note, it has been reported that a paretic propulsive reserve exists in chronic post-stroke patients [16,17,18]. Thus, rehabilitation schemes that aim to increase paretic propulsion, which is mediated by the plantar flexor during the late stance phase, are of growing interest. In order to increase plantar flexor functions, high-intensity training has been shown to be effective. This was demonstrated by increasing the resistive force during gait which elicited higher paretic propulsion when the resistive force was removed [16]. Also, incline gait on split belt treadmills has shown to increase paretic propulsion [17]. Swaminathan et al., demonstrated with an ankle targeted exosuit that resistance training increases the plantar flexion torque during gait [18]. Furthermore, investigations with exoskeletons have revealed some interesting results [19,20]. Together, these results suggest high dosages of task-specific volitional training of the plantar flexors are vital for promoting post-stroke gait rehabilitation.

A popular method for facilitating volitional movement in the paretic limb is constraint-induced movement therapy (CIMT), which promotes the usage of the paretic limb by restricting unafflicted limb movement [21,22]. This rehabilitation method has been shown to be an effective means for the recovery of post-stroke motor functions and has been widely utilized for upper-limb rehabilitation [23,24,25,26,27,28,29,30]. While there have been studies that investigate the effect of upper-limb CIMT on gait characteristics, to our knowledge, there has yet to be a direct lower extremity CIMT (LE-CIMT) application for gait rehabilitation because both limbs are necessary to perform gait.

**Figure 1:**
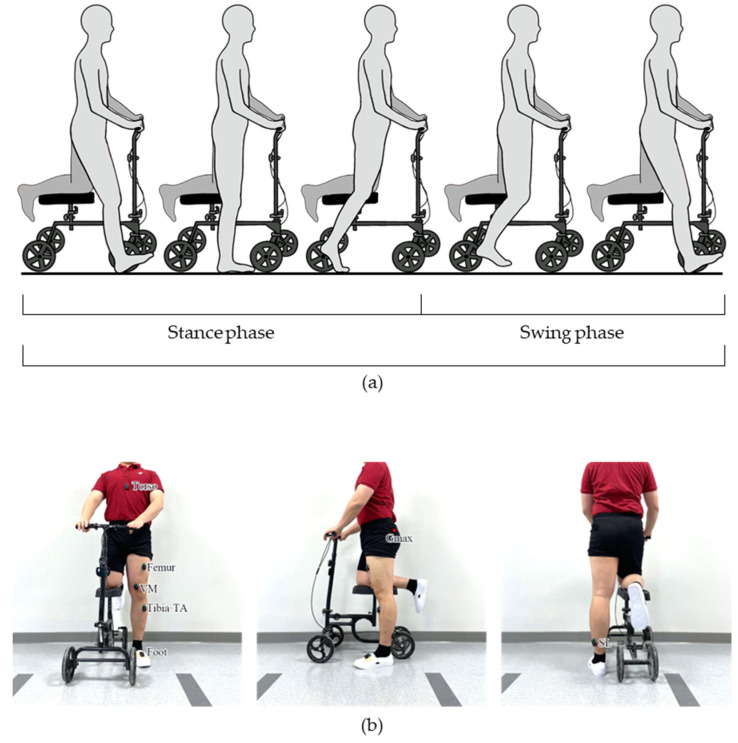
The knee scooter used in this study. (**a**) An illustration of gait using the knee scooter according to gait phase. (**b**) The *EMG* and IMU sensor placement positions.

In this study, we explore the feasibility of utilizing a knee scooter (Figure 1) as a practical device for LE-CIMT gait training, with a primary emphasis on the ankle plantar flexors that have a significant role in propulsive force. Conceptually, the unafflicted limb (inactive) would be placed on the knee scooter and the afflicted limb (active) would be used to propel the knee scooter forward. This would restrict the function of the inactive limb while providing an environment where the active limb can focus more on forward propulsion with less burden of weight support. We hypothesized that if the plantar force is reduced while gait-specific lower limb patterns are elicited through the usage of a knee scooter with able-bodied subjects, this method could be applied for LE-CIMT gait rehabilitation of stroke patients. The active lower-limb joint angles, plantar force, *EMG* patterns, stride length, and step time of movements with a knee scooter were compared with those of the natural gait. The results, gathered from a study involving 13 healthy subjects, show that the plantar force decreased and the gait patterns of the active limb using the knee scooter closely resemble that of natural gait, with minimal compensation patterns.

## 2. Materials and Methods

### 2.1. Participants

A total of 13 healthy volunteers (5 females and 8 males, age: 24.9 ± 4.9 years, height: 172.6 ± 9.0 cm, weight: 68.2 ± 9.5 kg) participated in this study. Those who had sustained lower-limb injuries in the past 6 months, had severe medical conditions or a history of such conditions, and individuals with cognitive impairments were not eligible to participate in this study. Before the start of our experiments, all participants provided written informed consent, and the research followed the ethical principles for human experiments as outlined in the Declaration of Helsinki, with its protocols approved by the Institutional Review Board of Korea University (IRB No. 2022-0399-01).

### 2.2. Experimental Setup

In order to assess the biomechanical effects of knee scooter during gait, participants were asked to perform 8 m of overground walking. Prior to data collection, the participants were given ample time to become familiarized with ambulation with the knee scooter. Once the participants felt comfortable, a total of 8 data acquisition sessions, 4 trials for 2 overground gait conditions: without the knee scooter at a self-selected slow speed (baseline) and with the knee scooter at a self-selected speed. Prior to the knee scooter trials, all subjects were instructed to stand up straight with both hands on the handles and with the right knee elevated on the knee scooter. Then, the height of the seat and handles were adjusted so that the upper body was upright, and the height of both knees was level (Figure 1b). The brake of the knee scooter was preset so that the wheels did not move during the swing phase. During the experiments, the subjects were instructed to move the knee scooter with the active leg as one would normally walk.

The joint angles, *EMG* activation patterns, plantar force, and stride lengths were acquired via IMU sensors (Delsys Inc., Natick, MA, USA), *EMG* sensors, PedarX (Novel GmbH, Munich, Germany), and Azure Kinect (Microsoft Inc., Redmond, WA, USA), respectively. The *EMG* data were obtained from the gluteus maximus (Gmax), vastus medialis (VM), tibialis anterior (TA), and soleus (SL) (Figure 1b). For all experiments the right limb was placed on the knee scooter and the left limb was designated as the active limb.

### 2.3. Data Acquisition

We only analyzed the sagittal joint angles of the hip, knee, and ankle which were estimated via 3D orientation data provided by the IMU sensors [31]. The relative orientation of the sensors that were placed on the opposite side of the joint was used to estimate the joint angle (Figure 1b).

Because direct comparison of the raw *EMG* signals is difficult, due to the high variability, the *EMG* waveform length (*WL*) was used to compare muscle activation patterns. In order to calculate the *EMG WL*, we first applied a band pass filter (4th order Butterworth; 20 to 500 Hz) to the raw *EMG* data, and then obtained the waveform length through Equation (1):

(1) $$EMG\;WL\left(n\right)=\sum_{i=n-N+2}^{n}\left|EMG\left(i\right)-EMG(i-1)\right|$$

where *n* is the current sample and *N* is the window size.

Facilitating appropriate plantar force sensitivity during task-specific training is vital because it serves as direct biofeedback to the user and has been previously reported to be closely associated with balance and the risk of falling [32,33,34,35]. Thus, it is imperative that we compare the foot pressure patterns during walking with and without knee scooters. The subject’s plantar force for the active limb was acquired using Pedar-X insoles (50 Hz), which contain a matrix of 99 capacitive pressure sensors that span the entirety of the insole. Furthermore, the anterior–posterior center of pressure (CoP_ap_) was evaluated, which is provided by the Pedar-X insoles.

In order to analyze the *EMG WL*, lower limb joint angles, and plantar force data according to the stance and swing phase, we first need to synchronize the data. The *EMG WL* and lower limb joint angles are already synchronized because they are acquired from the same sensors and the plantar force data are synchronized by applying an impact force to the bottom of the foot which is simultaneously detected by the foot pressure insole and the IMU sensors placed on the SL. Then, epochs such as heel contact and toe-off need to be identified which was executed by applying a simple threshold on the plantar force. The synchronized data were then segmented into gait cycles via the heel contact events and the segments were further divided into stance and swing phases using the toe-off events. 

**Figure 2:**
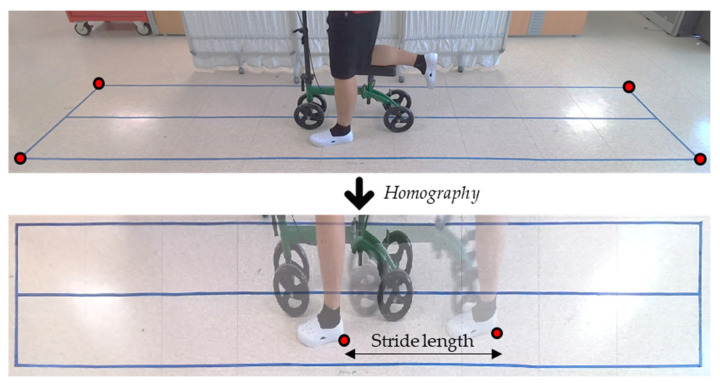
Input image and top-view image of the floor generated by homography matrix.

To acquire the stride length, we analyzed the video acquired from an RGB camera and applied an algorithm that is similar to that of Kentaro et al. [36]. This method consists of two steps: first, estimating the homography matrix to map the images to the ground plane; second, manually finding the frame number at heel contact and toe off so that we can estimate the stride length and step time (Figure 2). The ratio of the stance and swing phase within the gait cycle was measured using plantar force data.

### 2.4. Data Processing

We would like to present the common characteristics that were observed across the participant group; thus, we performed a group analysis of all participants. A means to normalize the individual’s *EMG WL* patterns and data is needed. Because raw *EMG* data acquired from different subjects cannot be directly compared, we normalized the *EMG WL* signal to the peak *EMG WL* value observed during natural gait. The plantar force data were normalized for each subject by representing the data in percentages of bodyweight. And the CoP_ap_ was normalized to the length of the insole. Once the data were normalized, the median data for each subject was acquired and the group data were presented as the median, 25th, and 75th percentile. A custom MATLAB (Mathworks Inc, Natick, MA, USA) script was used to perform all data processing.

## 3. Results

### 3.1. Joint Angles

**Figure 3:**
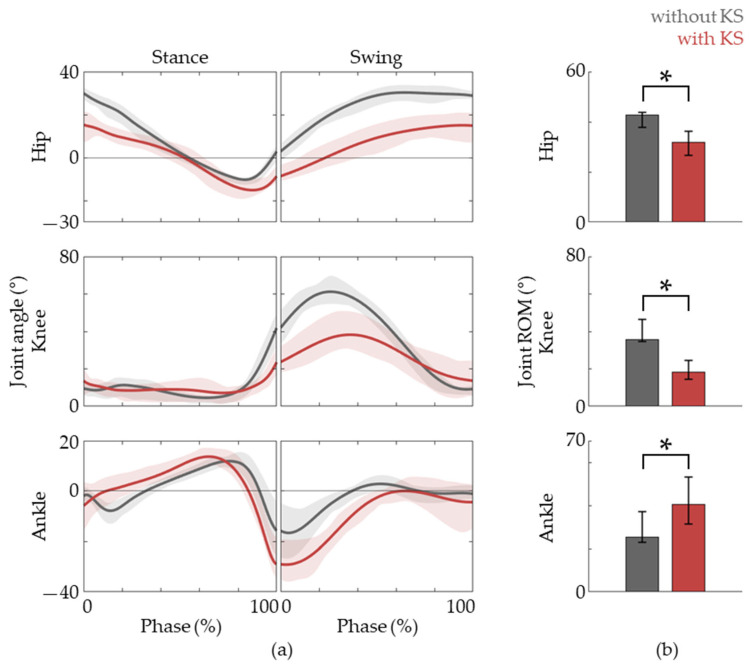
Joint angle data. (**a**) The hip, knee, and ankle joint angles during the gait phases, the baseline (gray lines) and with the knee scooter (red lines) are shown. (**b**) The ROM of each joint during the gait cycle. The lines are represented as medians, and lower and upper bounds of the shaded areas represent 25th and 75th percentiles, respectively. The asterisk (*) means *p* < 0.05, assessed using the Wilcoxon’s signed-rank test.

In order to assess whether the kinematic patterns of the lower-limb match that of natural gait well, we analyzed the hip, knee, and ankle joint angles according to the gait phases (Figure 3). While there were some differences, we found that the overall joint angle patterns during gait using a knee scooter were similar to that of natural gait. In detail, there is less hip flexion throughout the gait cycle, less knee flexion during the swing phase, and more ankle plantar flexion during the late stance to early swing phase. These observations can be seen in the joint angle traces shown in Figure 3.

### 3.2. EMG

**Figure 4:**
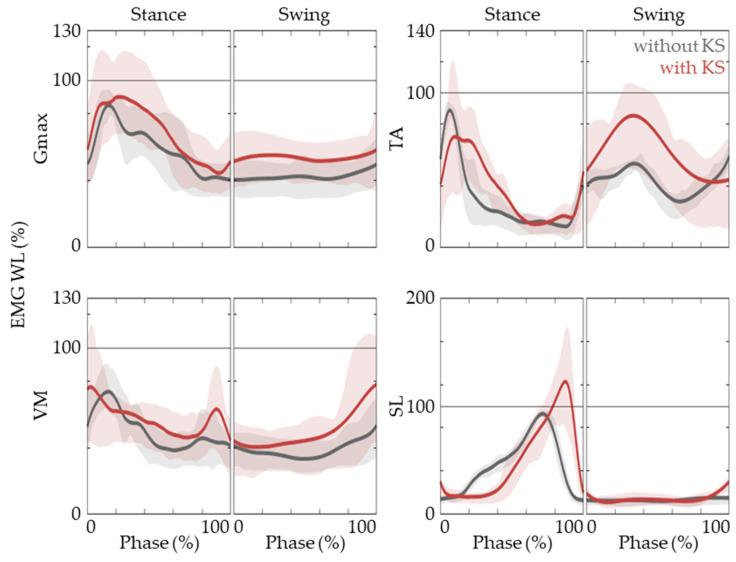
*EMG* activation patterns of Gmax, VM, TA, and SL. The *EMG WL* time traces are shown while gait without (black lines) and with (red lines) a knee scooter. The lines are represented as medians, and lower and upper bounds of the shaded areas represent 25th and 75th percentiles.

In order to promote functional recovery, it is vital for the muscle activation patterns of the task-specific training to match that of natural gait. This implies that not only each joint motion but also the muscle *EMG* patterns of gait with the knee scooter need to agree well with the baseline. Figure 4 shows that the Gmax, TA, VM, and SL *EMG* activation patterns of the active limb align well with that of natural gait. These muscles are reported to be responsible for the hip, knee, and ankle joint movements. It is also observed that TA activation in the early stance phase is prolonged and there is a delay in SL activation. 

### 3.3. Plantar Force and Spatio-Temporal Data

**Figure 5:**
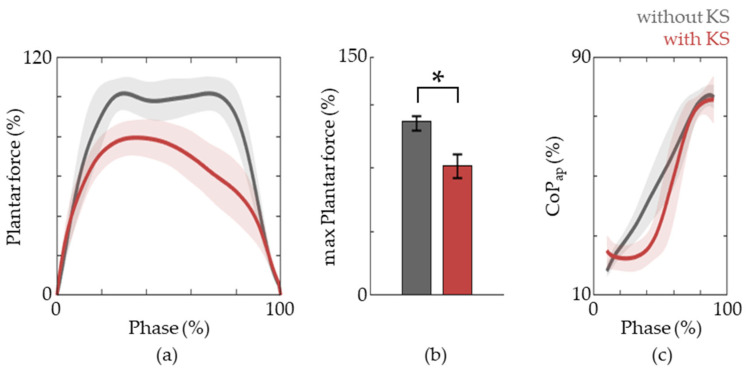
Plantar force patterns. (**a**) The plantar force during the stance phase of gait without (grey lines) and with (red lines) a knee scooter. (**b**) The maximum plantar force during the stance phase. The data are represented as medians, and 25th and 75th percentiles, respectively. The asterisk (*) means *p* < 0.05, assessed using the Wilcoxon’s signed-rank test. (**c**) The trajectories of CoP_ap_ for gait with and without the knee scooter are shown.

We next investigated the plantar force and CoP_ap_ of the active limb. During natural gait, the stance phase is initiated via heel contact and progresses through flat-foot and then toe off. Thus, CoP_ap_ starts from the heel and moves forward towards the toes. This is vital to facilitate the heel, ankle, and forefoot rocker functions. Thus, the training of these patterns is crucial and the importance is moreover emphasized because the foot is the only part of the body that comes in contact with the ground and provides direct feedback to the patient via plantar force sensitivity. The results show that this pattern is also observed in the active lower limb when riding a knee scooter (Figure 5). Moreover, we observe ~20% decrease in peak plantar force which alleviates the burden for weight support so that training can be focused on the generation of forward propulsion.

**Figure 6:**
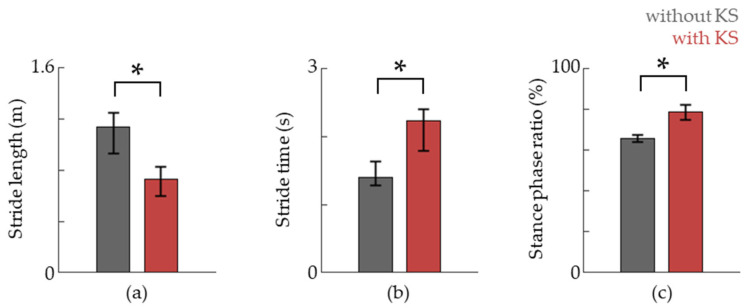
The stride length, stride time, and stance phase ratio are shown in (**a**–**c**), respectively. Data are shown as the median, and 25th and 75th percentiles, respectively. The asterisk (*) means *p* < 0.05, assessed using the Wilcoxon’s signed-rank test.

We also evaluated the stride length, stride time, and stance phase ratio via RGB camera analysis and plantar force data (Figure 6). The stride length was reduced compared to that of natural gait while the step time as well as the relative time spent in the stance phase was increased (Figure 5). This suggests that more emphasis is given towards stance phase-specific tasks.

## 4. Discussion

In this work, with able-bodied participants, the feasibility of a potential LE-CIMT method for post-stroke gait rehabilitation was tested. We found that the lower extremity kinematics elicited while using the knee scooter match that of natural gait well. Moreover, the *EMG* patterns suggest that the lower-limb muscles are activated according to the gait phase while plantar forces were reduced throughout the stance phase. This suggests that, for the active limb, gait-specific tasks can be trained with a lower burden of supporting weight. Altogether, these preliminary observations demonstrate the viability of our prosed gait rehabilitation method for hemiplegic gait.

We did observe some differences between gait with the knee scooter and that of the baseline. For example, the ROM for the hip and knee decreased while there was an increase in ankle ROM. Also, a delayed and increased activation of the SL was observed. Furthermore, not only the duration of the stance phase increased but also the stance-to-swing ratio. While it has not been tested with actual patients, these differences might actually be beneficial for LE-CIMT gait training because forward propulsion using a knee scooter is facilitated more by the ankle compared to that of natural gait. Also, the increased stance time would allow the user to prepare and facilitate plantar flexor activity.

Previous studies have shown that resistive training can bring about the underused propulsive reserve in the paretic limb. According to these studies, post-stroke patients possess a latent propulsion reserve and increasing the demand levels during training can elicit positive rehabilitative outcomes [16,37,38]. Evidence has shown that participants indeed possessed a sufficient reserve to generate more propulsion than what was initially observed with unrestricted gait. While it was not implemented, our LE-CIMT gait training method can easily adopt variable resistive forces by adjusting the friction of the wheels via a simple variable braking system. This would allow appropriate demand accommodations for not only various patients, but also throughout rehabilitative progressions.

In regard to forward propulsion, one must consider the trail limb angle (TLA). If ankle plantar flexion occurs too early, when the foot is either directly below or more forward than the pelvis, there would be no net forward propulsion. Thus, it is critical for plantar flexion to occur in the late stance phase. Thus, the delayed activation of the SL and plantar force, compared to that of natural gait, can be considered to be a desirable reaction when using the knee scooter as a post-stroke rehabilitation device. Furthermore, the knee scooter could be utilized for the training of post ankle surgery patients.

While this work showed promising results, we note that there exists a critical limitation, as only able-bodied subjects participated in this study. Also, one concern is that the new task of creating propulsive forces via the active limb, paretic in cases of patients, could be redistributed across the limb rather than targeting the ankle. Nevertheless, this study presents itself as a proof-of-concept which is necessary prior to further research with actual patients. These findings may have important implications for a new method of gait rehabilitation as CIMT can be applied to functional gait recovery.

## Data Availability

The data presented in this study are available on request from the corresponding author.
